# Dental Undergraduate Views of Objective Structured Clinical Examinations (OSCEs): A Literature Review

**DOI:** 10.3390/dj4010006

**Published:** 2016-03-19

**Authors:** James Puryer

**Affiliations:** School of Oral and Dental Sciences, Bristol Dental Hospital, Lower Maudlin Street, Bristol BS1 2LY, UK; james.puryer@bristol.ac.uk; Tel.: +44-(0)117-342-4184

**Keywords:** dental undergraduate, student, objective structured clinical examination, OSCE, assessment, views, anxiety

## Abstract

Objective Structured Clinical Examinations (OSCEs) are widely used in dental undergraduate assessment, often determining progression or graduation. Student evaluation of this assessment process is important, and this includes identifying the views of the student. The aim of this paper is to present a review of the current literature regarding dental student perceptions of OSCEs. A search of the PubMed database covering the period 1975 to 2015 identified 121 possible papers from which only six were suitable for review. The remaining papers were excluded due to them not reporting on dental undergraduate views. Students perceived the OSCE to be a valid assessment in three studies, but not in one. The educational benefit of an OSCE is well supported by these studies. OSCEs can induce high levels of anxiety compared to other forms of assessments, but this did not affect student performance. The majority of students would chose to have a similar format of assessment again, showing support for OSCEs. Further research using larger cohorts of students could be undertaken in order to support these finding which would give added evidence for the continuing use of OSCEs as a valid method of both dental undergraduate education and assessment.

## 1. Introduction

Objective Structured Clinical Examinations (OSCEs) were first described in 1975 [1], and following their use in medical assessments, the format was introduced into dental education [2,3,4]. It was soon suggested that an OSCE was “the gold standard for clinical assessment” [5].

OSCE have many advantages over traditional “long case” clinical assessments including being able to more easily control both the variables and complexity of the examination, being able to clearly define its aims to both teaching staff and students, and being able to assess a wide range of the students’ knowledge and competencies. This can result in a reliable overall view of the clinical competence of students being made [1]. Areas of performance essential to healthcare professionals, such as the ability to interpret data, problem solve and communicate with patients can be tested more readily than with traditional clinical examinations [6]. In addition to testing a student’s competence, an OSCE can be used to assess students beyond simply recollection of facts, and can move into higher orders of cognition. This is in preference to traditional multiple-choice examinations which, although can determine a student’s ability to recall information and principles, are not ideal at assessing higher levels of thinking [7].

An OSCE can be considered a “fair” assessment as all students face the same clinical scenarios, are assessed by the same examiner at each station, and the use of trained actors rather than “real” patients can help to maintain consistency for each student. This also helps minimise subjective bias. The increased objectivity by removing patient and examiner variation should help to ensure that the only variable being assessed is the ability of the student. A high degree of inter-examiner reliability in an OSCE assessment has been found [8].

The use of standard setting methods helps to ensure that decisions are based on non-arbitrary explicit criteria, which are combined in a systematic, reproducible, objective and defensible manner [9,10]. An OSCE also allows absolute standards to be set (criterion referencing) rather than allowing relative standards to be set (norm referencing). Absolute standards are expressed in terms of the performance of students against the assessment material, and do not compare the performance of one student against others taking the same assessment. Students will either “pass” or “fail” depending upon how well they perform. This is essential when assessing clinical competency, as it shows that a student has reached either a certain level of skill, or has acquired an agreed level of knowledge. OSCEs can be standard set by various methods, but this is usually carried out by the “Angoff” method or by a “Borderline Regression” method [11,12]. Both methods are widely in dental assessments, but there is growing evidence that the Borderline Regression method is more appropriate [13,14,15]. Prior to the OSCE taking place, the expected criteria of each station can be made explicit to both staff and students.

Whilst there are many advantages to using the OSCE as an assessment method, there are also potential disadvantages. From an educational perspective, an OSCE will only assess a student’s, knowledge, skill and ability in small sections, rather than in a “holistic” manner of patient care and treatment. In addition, it could also be argued that the format of the OSCE, namely assessing specific defined skills, favours assessment reliability whilst reducing validity. However, the OSCE can be combined with other forms of assessment, such as observation of student treatments on clinic to determine additional competencies.

There have been concerns regarding examiner fatigue, especially if multiple rotations of the OSCE circuit are carried out within the same day [16], although a subsequent study found no evidence of this [17]. It has been suggested that is the repetitive nature of the observations, usually requiring examiners to play close attention, but often with no student interaction that contribute to this. One possible solution to this is to allow examiners to change their stations between circuits, although this can reduce examiner consistency.

However, the greatest challenge to OSCEs are their intensive drain on resources, both in terms of equipment and staff time [18]. Staff are needed to both develop and pilot new OSCE stations before they are used in summative assessments for the first time [19], and also to physically set up and run the OSCE, being utilised for roles such as timekeepers, marshals to direct students between stations, staff and student briefings. There are obvious financial implications of this.

For any assessment method, it is crucial to evaluate this assessment process in order to maintain quality and confidence in the assessment process, and this should include the views of the student [20]. This is especially relevant to withstand possible legal challenges as many undergraduate OSCEs influence student progression or graduation. Evaluation of the assessment process can allow academic staff to modify and improve teaching and communication for future OSCEs, thus ensuring that it remains a valid and fair assessment of undergraduate clinical skills, and one that students are fully prepared for. The assessment process will become more transparent and will hopefully be seen by students as an aid to learning with educational value, rather than one that is trying to “catch them out” [19]. This is particularly important within the United Kingdom as undergraduate students are asked annually to participate within the National Student Survey (NSS), the results of which will be used to rank dental schools. One of the key themes of the NSS is student satisfaction levels relating to assessment methods and feedback. An improvement in student satisfaction will ultimately improve the ranking of the school. Thus there are benefits to students, patients and teaching institutions in developing quality assurance of assessments.

There is a wealth of evidence exploring medical undergraduate views of OSCEs, but there appears to be little knowledge of the views of dental undergraduates. The aim of this paper is to present a current overview of dental undergraduates’ subjective general perceptions of OSCEs as an assessment method through a review of the literature.

## 2. Method

The PubMed database was used to search for relevant papers covering the period 1975 to 2015, and various combinations of keywords were used to focus the search. Eligible for inclusion in this review were papers published in English, with no exclusions imposed by country of origin. A total of 121 papers were identified and the distribution of articles according to the combinations of key terms are shown in Table 1.

Following this initial search, 118 articles were discarded as either their titles or abstracts indicated they did not provide original information regarding undergraduate views or opinions about OSCEs. As only three papers were left for inclusion in the review, these papers’ references were studied to source other possible papers for inclusion and this generated a further three papers. Six papers were therefore included in the final analysis (Table 2), and the full texts of these papers were reviewed.

## 3. Results

Various themes were drawn post-hoc from this literature review regarding dental undergraduate views on OSCE assessments. These themes were identified as the emerging “key themes” from the six studies.

### 3.1. Subjective Validity and Reliability

The UK study compared and contrasted different types of clinical skills scenarios within an OSCE, and students generally disagreed that their operative skills were being validly tested [3]. Students were also sensitive to the assessment limitations, and the three main limitations identified were that firstly, there was a lack of clinical authenticity, secondly, there was a lack of communication skills testing, and finally, there was a lack of assessment of patient management. Students were doubtful that of the validity of the use of phantom heads in assessing clinical skills. In contrast, the Indian study found that the OSCE was a valid and reliable form of assessment [21]. Additionally, the students concluded that it was a “meaningful examination” and was a fair assessment due to all students being examined uniformly. The students also found the marking to be both transparent and objective [21]. Support for the validity of the OSCE was found in the Jordanian study [22] where 70% of students thought that the OCSE was objective, and significantly, 65.8% (*p* < 0.001) though that it was a good test of clinical skills. The general consensus was that the OSCE was a suitable format in which to test their operative dentistry, clinical judgment and skills, and 65.5% (*p* < 0.001) responded that it was a better evaluation of their clinical skills than other forms of assessment. The majority (72.3% *p* < 0.001) of these respondents would choose to have a similar format of assessment in future. However, some students (34.5%) were concerned that the OSCE did not effectively measure their clinical skills and was just another theoretical test, supporting the findings of the earlier UK study. Further support for the validity of the OSCE format comes from the most recent USA study where an identified theme found that the OSCE was an authentic assessment that required integration and application of knowledge [23].

### 3.2. Subjective Educational Benefit

The UK study found that when an OSCE circuit comprised “diagnostic” scenarios, 100% of students thought that the assessment was a useful educational exercise, although this dropped to 51% when the circuit comprised “operative” scenarios [3]. The Danish study found a very positive view of the OSCE from both candidates and examiners [24]. The majority thought that the format of the OSCE was relevant, was beneficial for both education and assessment, and was able to improve student learning, giving them “a very good impression of their own strengths and weaknesses” [24]. Encouragingly, all teachers found that the OSCE identified aspects of teaching that needed change [24]. The education benefit of OSCEs is supported by the Indian study where it was found that 89% of students could identify their weak areas, and 63% of students felt motivate to learn further [21]. The provision of feedback as part of a dental OSCE was a feature of this study and this was favoured by both students and examiners [21]. Further support comes from the most recent USA study where it was found that student perceptions were positive [23]. Students agreed that the OSCE went beyond memorization of facts, required application of knowledge, required the ability to think critically and problem solve, assessed clinically relevant skills and was a learning experience. Overall, students found the OSCE to be an authentic assessment and a learning experience [23]. The qualitative part of this study produced findings with the following themes [23]:

- The experience of taking an OSCE is different from that of taking traditional examinations because it requires greater integration and application of knowledge;
- The OSCE presents questions applicable to real-world clinical situations in an objective manner;
- Courses that involve case-based, small group discussion best prepare students for an OSCE;
- Feedback about performance on an OSCE is important for making the examination a valuable learning experience.

### 3.3. Anxiety Levels

The Dutch study concluded that the OSCE was the most anxiety-provoking method of assessment, and also that the students undertook greater preparation for the OSCE than they did for other assessment formats [25]. However, the study also found that the reported levels of student anxiety were not predictive of their OSCE results. Anxiety amongst students was also found in the Indian study where 63% felt that the OSCE format was more stressful than traditional formats of assessment, and 79% claimed to be “frightened/scared” when performing in a faculty member’s presence [21].

## 4. Discussion

This literature review shows that, in general, dental undergraduate students have positive perceptions of OSCEs. However, due to the low number of studies available and their small size, questions about the generalisability of the results must be raised. When looking at student perceptions of OSCE validity, it must be remembered that the students are reporting “subjective validity” as none of the studies tested validity in a psychometric sense. Students’ perception of subjective validity was found in three of the studies [21,22,23] but not in one study [3]. Where it was not found, this was primarily due to students questioning whether the use of dental manikins was a valid test of their clinical skills and communication skills. In 1990, Miller described a conceptual “pyramidal” model to show the various facets of clinical competence. [26] Students need to *know* the facts that underpin clinical practice, and also *know how* to apply these facts into their clinical practice. Furthermore, students need to be able to *show how* they would carry out clinical procedures based upon their knowledge. This *shows how* layer of the pyramid is important as it demonstrates a behavioural facet of clinical competence rather than just cognitive knowledge. The highest facet described by Miller is *does* which would allow students to demonstrate their clinical competence in real clinical practice. An OSCE is only able to assess the *shows how* level rather than the *does*, thus in order to fully assess the clinical competency of an individual, longitudinal assessments and “triangulation” of assessment methods are needed. Whilst the use of manikins may be needed to test some technical skills, students were aware of lack of clinical authenticity of these exercises. [3] The increased use of simulated patients/actors may help increase students’ subjective validity and authenticity, especially in areas where communication is vital such as with history taking, consent taking and discussion of treatment options, enabling an OSCE to more validly assess these non-technical skills. This development will help to raise the OSCE from a *shows how* level to a *does* level in Miller’s pyramidal of assessment of competence.

The subjective educational benefit of OSCEs is supported well by these studies. Students generally acknowledged that an OSCE highlighted areas of their strengths and weaknesses, and that they learned from the assessment, supporting the findings of others [27,28]. This was particularly so where student feedback on performance was given. The OSCE can provide a focus for useful and relevant learning, due to it conveying strong messages to students on what should be valued in terms of curriculum and learning outcomes. There can be a different influence of an OSCE and written examinations on students’ learning outcomes, and OSCE stimulated learning can give students a greater level of realistic self-assessment [29], and there is good support for the use of simulated scenarios within medical education. [30] In addition, an OSCE can provide “formative evaluation” as the student is actively participating within the assessment [31]. An OSCE can help to identify areas of teaching that are in need of change, and this has previously been carried out successfully. For example, The University of Medicine and Dentistry of New Jersey changed the format of their existing medical OSCE to that of a “teaching OSCE” in order to provide formative feedback to students following a standardised patient interaction [32]. Similarly, students from Harvard Medical School were found to perform better in some subject areas than in others when assessed by an OSCE, which led to a review of teaching techniques and the curriculum [33].

The Dutch study [25] was set up to specifically investigate anxiety levels among dental students when faced with different types of assessment methods, including OSCEs. It has previously been documented by various authors that examinations and assessment procedures have the potential to be both anxiety provoking and stressful for dental undergraduates [34,35]. The Dutch study concluded that the OSCE was the most anxiety-provoking method of assessment, and also that the students undertook greater preparation for the OSCE than they did for other assessment formats. However, this increased preparation time may give support to an OSCE supporting student learning. These results add to the evidence from other studies in allied medical disciplines where higher levels of anxiety have been found with OSCE assessments [36,37,38,39]. There is also anecdotal evidence that some dental students suffer from shaking hands and raised blood pressure when faced with an OSCE [40]. It has been suggested that these reported higher levels of anxiety may be due to the constant monitoring and observation by examiners during an OSCE [37], and also the timed, interactive aspects of the assessment [41]. However, it must be remembered that dental assessments in general (not only OSCE assessments) have been shown to produce psychological stress leading to possible burnout and mental health issues [42]. It is encouraging, that the Dutch study found that the reported levels of student anxiety was not predictive of their OSCE results, suggesting that despite OSCEs being the most anxiety-provoking method of assessment, this was no reason not to continue using them as an assessment method for dental undergraduates.

## 5. Conclusions

The majority of dental undergraduate respondents in these studies would choose to have a similar format of OSCE assessment in future, which gives strong student support for this type of assessment compared to traditional forms of assessment. Despite OSCEs invoking higher degrees of student anxiety when compared to other forms of assessment, this does not appear to affect performance. Further research, using larger cohorts of students, could be undertaken in order to support these finding which would give added support for the continuing use of OSCEs as a valid method of both dental undergraduate education and assessment.

## Figures and Tables

**Table 1:** The results of the initial search by pairs of keywords.

Keyword Pairs Used	Number of Citations
“Student” & “OSCE”	5
“Student” & “Objective Structured Clinical Examination”	26
“Undergraduate” & “OSCE”	17
“Undergraduate” & “Objective Structured Clinical Examination”	17
“Dentistry” & “OSCE”	15
“Dentistry” & “Objective Structured Clinical Examination”	1
“Dental” & “OSCE”	20
“Dental” & “Objective Structured Clinical Examination”	7
“Views” & “OSCE”	2
“Views” & “Objective Structured Clinical Examination”	1
“Opinion” & “OSCE”	0
“Opinion” & “Objective Structured Clinical Examination”	0
“Perceptions” & “OSCE”	5
“Perceptions” & “Objective Structured Clinical Examination”	5

**Table 2:** The studies included within the literature review.

Reference	Country	Student Year	No. of Students	Data Collection
Mossey *et al.* (2001) [3]	UK	4th Year	101	Questionnaire
Larsen & Jeppe-Jensen (2007) [24]	Denmark	3rd Year	68	Questionnaire
Brand & Schoonheim-Klein (2009) [25]	The Netherlands	3rd Year	89	Questionnaire
Lele (2011) [21]	India	3rd Year	19	Questionnaire
Hammad *et al.* (2013) [22]	Jordan	4th Year	134	Questionnaire
Graham *et al.* (2014) [23]	USA	3rd Year	78	Mixed-method
